# Expert proposal to analyze the combination of aortic and mitral regurgitation in multiple valvular heart disease by comprehensive echocardiography

**DOI:** 10.1007/s00392-023-02227-y

**Published:** 2023-05-22

**Authors:** Andreas Hagendorff, A. Helfen, R. Brandt, F. Knebel, E. Altiok, A. Ewers, D. Haghi, J. Knierim, N. Merke, E. Romero-Dorta, T. Ruf, C. Sinning, S. Stöbe, S. Ewen

**Affiliations:** 1 Department of Cardiology, University of Leipzig, Liebigstrasse 20, 04103 Leipzig, Germany; 2 Department of Cardiology, Kath. St. Paulus Gesellschaft, St-Marien-Hospital Lunen, Altstadtstrasse 23, 44534 Lünen, Germany; 3 Department of Cardiology, Kerckhoff Heart Center, Benekestr. 2‑8, 61231 Bad Nauheim, Germany; 4 Klinik Für Innere Medizin II, Kardiologie, Sana Klinikum Lichtenberg, Fanningerstrasse 32, 10365 Berlin, Germany; 5 Department of Cardiology, Angiology and Intensive Care Medicine, University of Berlin, German Heart Center Charité Berlin, Campus Mitte, Chariteplatz 1, 10117 Berlin, Germany; 6 Department of Cardiology, University of Aachen, Pauwelsstrasse 30, 52074 Aachen, Germany; 7 Department of Cardiology and Angiology, BG University Hospital Bergmannsheil, de La Camp-Platz 1, 44789 Bochum, Germany; 8 Kardiologische Praxisklinik Ludwigshafen-Akademische Lehrpraxis der Universitat Mannheim-Ludwig-Guttmann, Strasse 11, 67071 Ludwigshafen, Germany; 9 Department of Cardiothoracic and Vascular Surgery, German Heart Center Charité Berlin, Augustenburger Platz 1, 13353 Berlin, Germany; 10 Paulinenkrankenhaus Berlin, Klinik Für Innere Medizin Und Kardiologie, Dickensweg 25‑39, 14055 Berlin, Germany; 11 Department of Cardiology, Center of Cardiology, Heart Valve Center, University Medical Center Mainz, University of Mainz, Langenbeckstrasse 1, 55131 Mainz, Germany; 12 Department of Cardiology, University Heart and Vascular Center Hamburg, German Centre of Cardiovascular Research (DZHK), Partner Site Hamburg/Kiel/Lubeck, Martinistrasse 52, 20251 Hamburg, Germany; 13 Zentrale Notaufnahme and Klinik Für Innere Medizin III, Kardiologie, Angiologie Und Internistische Intensivmedizin, Universitätsklinikum Des Saarlandes, Homburg, Germany

**Keywords:** Echocardiography, Aortic valve regurgitation, Mitral valve regurgitation, Multiple valvular heart disease

## Abstract

The assessment of valvular pathologies in multiple valvular heart disease by echocardiography remains challenging. Data on echocardiographic assessment—especially in patients with combined aortic and mitral regurgitation—are rare in the literature. The proposed integrative approach using semi-quantitative parameters to grade the severity of regurgitation often yields inconsistent findings and results in misinterpretation. Therefore, this proposal aims to focus on a practical systematic echocardiographic analysis to understand the pathophysiology and hemodynamics in patients with combined aortic and mitral regurgitation. The quantitative approach of grading the regurgitant severity of each compound might be helpful in elucidating the scenario in combined aortic and mitral regurgitation. To this end, both the individual regurgitant fraction of each valve and the total regurgitant fraction of both valves must be determined. This work also outlines the methodological issues and limitations of the quantitative approach by echocardiography. Finally, we present a proposal that enables verifiable assessment of regurgitant fractions. The overall interpretation of echocardiographic results includes the symptomatology of patients with combined aortic and mitral regurgitation and the individual treatment options with respect to their individual risk. In summary, a reproducible, verifiable, and transparent in-depth echocardiographic investigation might ensure consistent hemodynamic plausibility of the quantitative results in patients with combined aortic and mitral regurgitation.

**Graphic abstract:**

The quantitative approach to assess LV volumes in combined AR and MR patients: explanation and algorithm of how to determine the relevant target parameters. LVSV_eff_—effective left ventricular (LV) stroke volume, LVSV_forward_—forward LV stroke volume through the aortic valve (AV), LVSV_tot_—total LV stroke volume, RegVol_AR_—regurgitant volume through the AV, RegVol_MR_—regurgitant volume through the mitral valve (MV), LV_filling volume_ = LV_MV_-Inflow − transmitral LV inflow, LVOT—left ventricular outflow tract, RF_AR_—regurgitant fraction of aortic regurgitation (AR), RF_MR_—regurgitant fraction of mitral regurgitation (MR), RVSV_eff_ —effective right ventricular (RV) stroke volume, RVSV_forward_—forward RV stroke volume through the pulmonary valve, RVSV_tot_—total RV stroke volume.

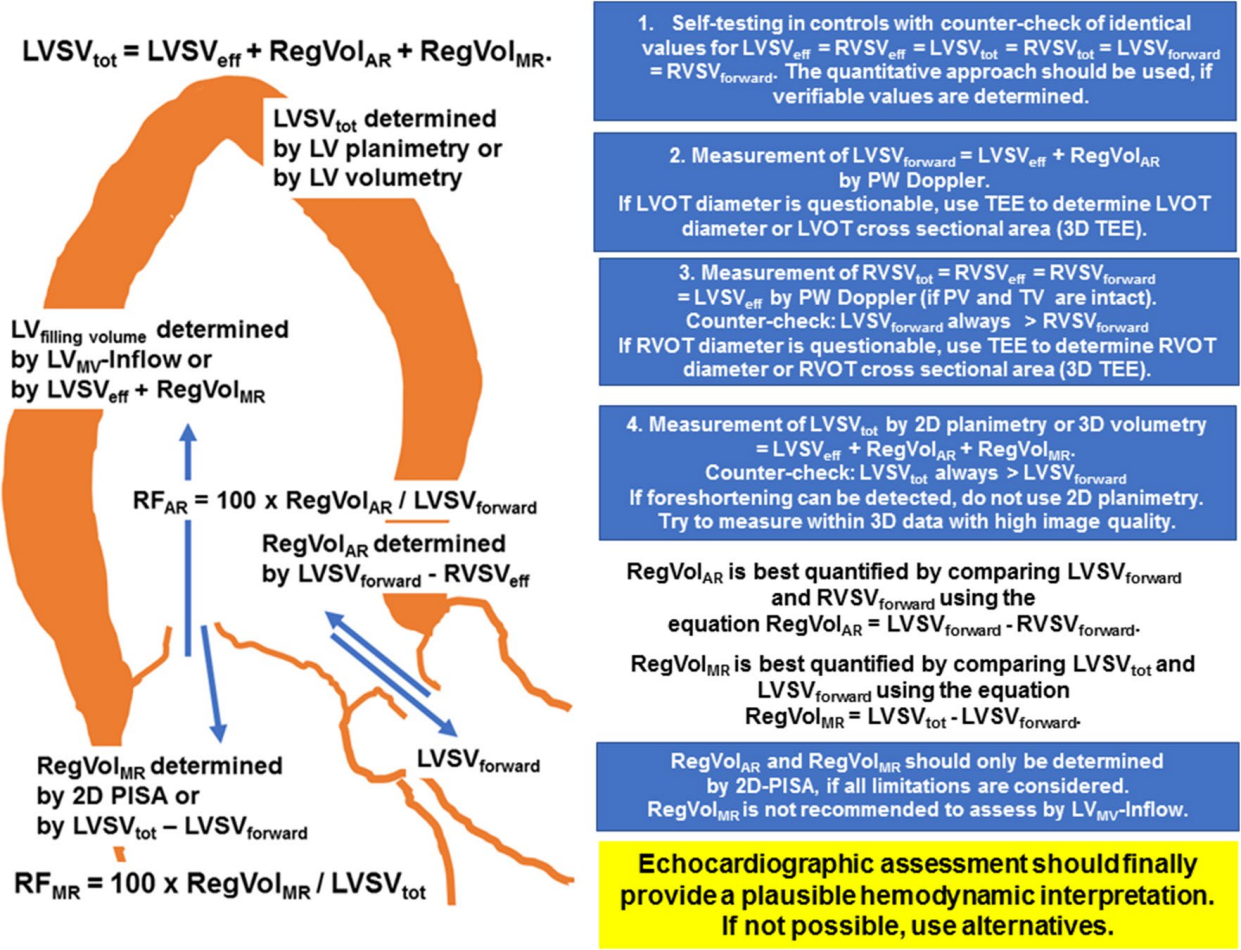

## Introduction

Multiple valvular heart disease (VHD) is defined as the presence of at least two concomitant valvular diseases of moderate or severe severity. In the Euro Heart Survey, the prevalence of multiple VHD is estimated as being up to 20%. However, the exact prevalence of combined aortic (AR), and mitral regurgitation (MR) remains unclear [1–3]. Combined AR and MR often share a common etiology due to degenerative, rheumatic, or acute inflammatory diseases [4]. Less commonly, they may result from two unrelated pathogenic entities [4].

The coexistence of AR and MR is a frequently encountered but understudied multiple VHD scenario [5]. It is important to characterize the respective mechanisms and severity of each lesion. Naturally, coexistent valvular disease carries a higher risk of mortality than a single lesion alone [6]. Both MR and AR cause left ventricular (LV) volume overload causing myocardial compensatory LV remodeling with the consecutive development of heart failure. Needless to say, the combination of both forms of regurgitation is worse than each regurgitation alone. The characterization of the mechanisms and degree of AR and MR in multiple VHD is of utmost importance, as it influences clinical decision making. The greater importance of one lesion and the interaction of both lesions must be considered for a timely judgment on therapy of either one, or both lesions. Echocardiography is the diagnostic method of choice in VHD [7]. While the integrative approach has been recommended for analysis of valvular regurgitations [8], its use may lead to incongruent findings [9, 10]. Hence, an exclusively quantitative robust method would be desirable.

Combined AR and MR often share a common etiology, with congenital valve defects, degenerative valve disease and rheumatic heart disease being the leading causes. Less commonly, combined AR and MR may result from two unrelated pathogenic entities [4]. An overview of different etiologies can be found in Table 1.

**Table 1:** Etiologies of combined AR and MR

Congenital valve defects
Degenerative valve disease
Rheumatic heart disease
Endocarditis
Marfan syndrome and related disorders (e.g., Loeys–Dietz syndrome)
Drug-induced VHD Anti-migraine drugs: ergotamine and methysergide Anti-Parkinson drugs: pergolide and cabergoline Anorexigens: fenfluramine, dexfenfluramine, and phentermine MDMA (3,4-methylenedioxymethamphetamine, commonly known as *ecstasy*)
Radiation
Rheumatoid arthritis and other connective tissue disorders
AR secondary to aortic root dilatation
MR secondary to LV and MV annulus dilatation due to chronic AR

*AR* aortic regurgitation, *LV* left ventricular, *MR* mitral regurgitation, *MV* mitral valve

### Pathophysiology and differences in the symptomatology of isolated AR and MR—explanation of different compensation mechanisms

The causes of AR and MR with their underlying mechanism are described according to the Carpentier’s classification of leaflet motion: Type I: normal leaflet motion, Type II: excessive motion, and Type III: restrictive motion [11]. Chronic isolated AR results in reverse transvalvular diastolic blood flow into the left ventricle due to primary (organic) damage of the cusps or secondary (functional) damage resulting from to dilatation of the aortic root complex causing a combined volume and pressure LV overload. Chronic AR usually evolves slowly and is well compensated in early stages, often documented by the presence of asymptomatic severe AR in physically fit patients [12]. Dyspnea upon exercise can usually be observed in early stages, whereas overt symptoms of heart failure like congestion, weakness, or arrhythmias occur in the later stages of the disease. The late appearance of symptoms in isolated AR due to effective compensatory mechanisms is explained mainly by the fact that both the left ventricle and the aortic root form part of the high-pressure system, separating the low-pressure system by an intact mitral valve (MV) protecting against damage to the left atrium, pulmonary vascular system, and right heart.. The volume overload in chronic AR results in progressive LV remodeling to normalize wall stress and maintain systolic function and is characterized by eccentric LV hypertrophy, LV dilatation, and LV spherification. Repetitive ischemic episodes caused by the ensuing increased LV end-diastolic pressure (LVEDP) are thought to promote myocardial fibrosis as the underlying mechanism for reduced LV compliance and diastolic dysfunction. LV dilatation and the reduction of LV ejection fraction (LVEF) as well as left atrial (LA) enlargement due to increased LV filling pressures in the later course of the disease [13–15] are prognostically unfavorable factors in chronic AR.

Chronic isolated MR results in reverse transvalvular systolic blood flow into the left atrium due to primary (organic) structural abnormalities of the leaflets and the MV apparatus or secondary (functional) damage resulting from eccentric LV hypertrophy, LV dilatation, annulus dilatation, and an imbalance between tethering and closing forces caused by pathological enlargement or geometric changes of the left ventricle or the left atrium [16–18]. Chronic MR usually develops slowly, but symptoms such as shortness of breath, edema, or palpitations appear earlier than in chronic AR.

The earlier onset of symptoms in isolated MR can be explained by the fact that the MV represents the boundary to the low-pressure system, and thus pressure elevation and signs of congestion in the pulmonary circulation are more likely to occur. In isolated MR, a considerable proportion of the total LV stroke volume (LVSV_tot_) empties into the low-impedance left atrium as mitral regurgitant volume (RegVol_MR_). To maintain the effective LV stroke volume (LVSV_eff_), which corresponds to the forward LV stroke volume (LVSV_forward_) in the absence of AR, both LV diastolic volume and LVSV_tot_ increase. During this early compensation, LVEF is usually in the normal-to-high range. This volume overload may lead to LV dilatation with eccentric LV hypertrophy and a change in the LV shape towards a more spherical LV cavity. Progressive LV remodeling may worsen or lead to secondary (functional) MR due to an imbalance between increased tethering forces and decreased closing forces during systole [19]. In the compensated stage, eccentric LV hypertrophy maintains a normal diastolic pressure with increase of wall stress due to predominant LV dilatation and with decrease of wall stress due to predominant LV wall thickening. LA enlargement is often a consequence of MR and may be associated with mitral annular enlargement inducing progression of secondary MR [20].

The combination of AR and MR may lead to LA and LV volume overload, which can result in atrial fibrillation, pulmonary hypertension, right ventricular (RV) enlargement, RV dysfunction and secondary tricuspid regurgitation in the low-pressure system. Importantly, RV dysfunction is a prognostic factor for postoperative mortality in patients with combined AR and MR [21, 22]. Currently, these coexistent pathophysiological consequences are given little consideration in the current guidelines [20]. The interdependency of AR and MR is based on the physical properties of incompressible fluid within the cardiac cavities. The cardiac chambers, therefore, serve both as a reservoir during filling and as a propagation pump during muscular contraction. When both forms of regurgitation are present, the reservoir function becomes the Achilles’ heel of LV remodeling due to LV volume overload caused by both relevant AR and MR. AR progression increases the forward flow through the AV as determined by Doppler echocardiography (LVSV_forward_), calculated as the sum of LVSV_eff_ and the transaortic regurgitant volume (RegVol_AR_). In the presence of AR and MR, eccentric LV hypertrophy serves as a compensatory mechanism to maintain an effective cardiac output. However, with LV dilatation and an almost unchanged LV wall thickness, LV wall stress increases inducing LV dysfunction.

### Clinical impact of combined AR and MR

In both AR and MR as a singular valvular lesion, the LV volume load increases. In contrast to MR, AR additionally increases LV afterload and thus causes an additional LV pressure load. Historical data show that in the presence of severe MR, mild-to-moderate AR may be well tolerated, but when AR is severe, any degree of MR may substantially worsen LV dilatation and LV dysfunction [23]. The safeguarding mechanism of early MV closure restricting the quantity of backward flow into the left atrium and pulmonary circulation in severe AR is not present in patients with combined AR and MR. This plays an important role in clinical worsening in comparison with isolated AR [6, 24, 25]. Postoperative data showed that LV dysfunction is more likely to occur in combined AR and MR than in isolated AR [26]. Another retrospective single-center study of 756 patients with at least moderate AR showed moderate to severe MR in 45%. Presence of moderate to severe and severe MR was associated with a larger LV size, lower LVEF, atrial fibrillation, as well as older age, female sex, and further comorbidities. Survival was increased if MR was also treated at the time of aortic valve (AV) replacement, and best if MV repair was feasible [27]. However, patients with combined AR and MR had a worse postoperative survival compared to patients with single-valve disease [6, 24]. Data on the diagnosis of combined AR and MR are limited in the literature [8, 11]. LVEF is recognized as a suitable variable to monitor LV function in VHD and can be used in combination with biomarkers like NT-proBNP to monitor potential impairment. Since LVEF is highly dependent on LV loading conditions, it has a limited ability to characterize abnormalities of myocardial contractility at early stages of severe combined MR and AR. Thus, LV deformation—especially global longitudinal strain—seems to be a more sensitive indicator of incipient LV dysfunction than LVEF [28]. For any given level of LV end-systolic volume, LV dysfunction is discussed as a prognostic marker in MR and AR [21, 22]. Severe LV dilatation may occur even in the combination of moderate AR plus moderate MR [5]. Consequently, the coexistence of significant AR and MR intensifies the negative impact on LV function and is associated with a worse prognosis compared with a single valvular lesion [3, 5, 24]. Moreover, the combination of non-severe AR and MR may lead to a clinically significant severe hemodynamic burden [22, 29].

For combined AR and MR, it often needs to be determined whether both valvular lesions or only one lesion are responsible for the pathological LV and RV changes [3, 5, 6, 20]. In a large cohort of 1239 patients with at least moderate AR, the incidence of at least moderate functional MR was 9%, and of primary MR 5%. Functional MR was associated with larger LV volumes and lower LVEF. The long-term mortality of AR patients was increased by concomitant MR—more so by functional MR than by primary MR [6]. In patients with moderate or severe AR, at least moderate functional MR was documented in 23%. Lower LVEF and a larger LA, as well as more MV tenting and larger interpapillary muscle distances were more frequently associated with MR [30].

### Importance of Doppler echocardiography to distinguish between non-valvular and valvular causes of LV dilatation due to combined AR and MR

The clinical relevance of combined AR and MR is usually obvious if one or both defects are moderate to severe. However, decision-making is rendered difficult if both defects are rated as mild to moderate, and symptoms of heart failure exist with no other obvious cause. Since cardiomyopathy of other causes is possible in the presence of LV dilatation, the differentiation from other non-valvular causes of heart failure is important [31]. AR contributes to delayed MV opening causing a prolonged isovolumetric relaxation time with LV filling due to AR prior to diastolic forward flow through the MV. Thus, LV filling pressure rapidly increases as a result of simultaneous LV filling due to AR and through the MV. The diastolic LA pressure is the driving force of LV filling. The effective regurgitant orifice area (EROA) of the AR serves as flow resistance of the diastolic pressure at the level of the tubular ascending aorta, which attenuates but does not abolish the diastolic driving forces of forward LV filling. Consequently, shortening of the pulmonary acceleration time (< 100 ms) and an increase in systolic pulmonary artery (PA) pressure occur in early stages of MR. In addition, shortening of transmitral E-wave acceleration and deceleration, and velocity reduction of the A-wave are signs or ‘red flags’ of relevant combined AR and MR. The restrictive transmitral LV filling pattern is in accordance with indirect evidence of reduced LV compliance or atrial cardiomyopathy in LA and/or LV dilatation.

### Potential echocardiographic presentation of the predominant component in chronic AR and MR

LV remodeling due to chronic AR is characterized by eccentric LV hypertrophy and LV dilatation attributable to chronic AV damage—for example, in case of cusp prolapse of a bicuspid AV—or by aortic annulus dilatation in pathologies of the aortic root complex [32, 33]. In patients with hemodynamically significant AR, functional MR due to LV remodeling is observed in approximately 7% and is considered a more advanced stage in the natural course of the disease. In AR, wall stress was found to be markedly elevated due to a markedly increased afterload, whereas in MR wall stress reached only near-normal levels [34]. For valve regurgitation of similar severities, AR results in greater LV dilatation to the point of irreversible myocardial dysfunction compared with MR [35]. Acute worsening of combined chronic AR and MR may occur as a result of reaching the compensation limits for regurgitant volume at both valves. Examples of additional acute components of valve destruction are acute valve infection (endocarditis) or acute ischemia (myocardial infarction—especially due to occlusion of the circumflex and marginal branches—causing partial or complete rupture of papillary muscles). Chronification of high-grade secondary MR is unlikely, since chronic symptoms are expected to occur in early stages of MR. Thus, MR developing secondary to chronic severe AR is a unique subtype of combined AR and MR. Its prevalence has been reported to be between 6 and 45% and its occurrence has been associated with chronic changes in the size, shape, and function of the LV [36]. However, despite significant increases in LV dimensions commonly assumed to be associated with secondary MR, such as LV volume, LV sphericity, tethering distance and mitral annular size, severe secondary MR may be rather rare in chronic severe AR [37]. This seems to be due mainly to the ability of the MV to increase its leaflet area and thickness, thereby counterbalancing the consequences of chronic AR [37]. This enlargement of leaflet area is thought to protect against MR and seems to be lacking or blunted in functional MR due to LV dilatation and LV dysfunction. These data were recently corroborated by a study in sheep where a serotonin inhibitor nearly abrogated the development of functional MR by intensifying mitral leaflet growth after induction of myocardial infarction [38].

Albeit the LV volume load increases due to MR, relevant AR secondary to severe MR seems unlikely. LV remodeling due to chronic MR can, in theory, cause AR due to aortic annulus dilatation. However, dilatation of the left ventricular outflow tract (LVOT) is rarely observed even in severely dilated left ventricles. Furthermore, the aortic annulus withstands tethering forces by the surrounding tissue and myocardium much better than the mitral annulus (MA). Assuming a linear progression and excluding patients with endocarditis and diseases of the aortic root complex, observational studies showed that, on average, chronic AR progresses within more than 25 years [39].

In conclusion, the most prevalent phenotype is the combination of AR with functional MR due to LV dilatation. MR per se does not lead to AR, and the simultaneous occurrence of (pure) primary AR and/or (pure) primary MR is rare but possible in the context of endocarditis.

### Problems of echocardiographic analysis of combined AR and MR

Concerning the grading of single valvular lesions in AR and MR, current guidelines suggest the use of an integrative approach with respect to methodological limitations (Table 2). [7, 8, 11, 20]. Regarding the echocardiographic evaluation of the combination of AR and MR, no specific recommendations exist that would fundamentally differ from the evaluation of single-valve regurgitant lesions [3, 40]. However, assessment by echocardiography should attempt to identify the hemodynamic scenario in which the combination of AR and MR becomes relevant. Thus, echocardiography in multiple VHD—especially in patients with combined AR and MR—is challenging due to the interdependency of both forms of regurgitation [3, 5, 8, 24, 36].

**Table 2:** Echocardiographic parameters for assessing AR and MR severity, individual cut-offs for severe AR and MR, and their limitations—especially in combined AR and MR

	Severe AR	Severe MR
Jet area	Only qualitative information	Only qualitative information
General limitations	The jet area, the ratio of AR jet diameter and LVOT diameter, the ratio of AR jet area and LV area, as well as the ratio of MR jet area and left atrial area are not recommended for grading the severity of regurgitation in the guidelines. The many confounding factors render these findings unreliable	
Monoplane vena contracta	> 6 mm	> 7 mm
General limitations	Not standardizable in variable, non-circular regurgitant orifice areas: not applicable and defined in multiple jet formations; extremely dependent on the sectional plane visualizing the central longitudinal jet formation; only feasible after optimizing image settings (no smoothing of pixel size, individually optimized 2D gain, color gain, color Doppler frequency, low velocity reject, frame rate)	
Biplane vena contracta	–	> 8 mm
General limitations		Not standardized in the literature
Doppler techniques	Pressure half time (PHT) by CW-Doppler < 200 ms Reversed end-diastolic flow velocity > 0.2 m/s, determined in the proximal descending aorta or the left subclavian artery	Maximum of transmitral E-velocity by PW Doppler > 1.5 m/s in the absence of other causes of elevated LA pressure and of mitral stenosis Tribouilloy ratio = mitral-to-aortic velocity time integral (VTI) ratio = VTI_transmitral_/VTI_LVOT_ by PW Doppler > 1.4
General limitations	Standardization of the sample volume of the PW Doppler is necessary; alignment of cw-Doppler angulation parallel to the corresponding jet direction is necessary. PHT is influenced by chamber compliance	
Special limitations	Both AR and MR affect diastolic LV properties in hard-to-predict ways. Thus, using the PHT of regurgitant flow velocities to grade AR severity is even less reliable in the presence of MR than in isolated AR	Transmitral E-velocity is unreliable in the presence of increased transmitral flow due to mitral stenosis; VTI_transmitral_/VTI_LVOT_ ratio cannot be used in the presence of relevant mitral stenosis and/or AR
EROA by 2D-PISA method	≥ 0.3 cm^2^	≥ 0.4 cm^2^ ≥ 0.3 cm^2^ if EROA is elliptical
General limitations	2D-PISA is reliable only in jet formation with an alignment parallel to the corresponding jet formation; it is error-prone in presumably non-circular orifice areas and highly error-prone in case of incorrect labeling of the PISA radius. 2D-PISA does not consider the dynamic nature of MR	
Special limitations	Presumably, 2D-PISA may be reliable only with right coronary cusp prolapse using the parasternal long-axis view—adequate alignment is not possible in all other AR jet formations	2D-PISA is not reliable and error-prone in eccentric jet formations, in multiple jets, in lesions like perforation or cleft, in tenting of the leaflets, and in primary MR
Regurgitant volume	≥ 60 mL	≥ 60 mL ≥ 45 mL in conditions of low flow
General limitations	The extent of regurgitant volumes depends on the extent of LV total stroke volume; therefore, the parameter of regurgitant volume must be interpreted with respect to LV effective stroke volume	
Special limitations	An individual cut-off for isolated severe AR cannot be used if the calculated LV effective stroke volume is in a range that is too low and not plausible	An individual cut-off for isolated severe MR cannot be used if the difference between LV total stroke volume and calculated regurgitant volume results in an LV effective stroke volume in a range that is too low and not plausible
Regurgitant fraction	≥ 50%	≥ 50%
General limitations	Individual assessment of the regurgitant fraction requires a reliable and verifiable assessment of cardiac volumes resulting in plausible hemodynamic scenarios. The estimation of the MR regurgitant fraction in combined AR by calculation of the LV total stroke volume is not reliable and error-prone	
Special limitations	In isolated AR, the regurgitant fraction can be assessed by measuring forward stroke volumes in the LVOT and RVOT. However, determining RVOT dimensions is challenging. The cross-sectional area of the proximal pulmonary trunk is easier to measure than the most often elliptical area of the RVOT. The assessment of LV inflow by transmitral VTI and mitral annulus dimension is highly error-prone. The assessment of regurgitant fractions using regurgitant volumes by 2D-PISA method is severely limited by methodological limitations	In isolated MR, the regurgitant fraction can be assessed by planimetric or volumetric measurement of LV total stroke volume and the measuring the LV forward stroke volume in the LVOT. The difference between both volumes represents the MR regurgitant volume which can be used for calculating the regurgitant fraction. The assessment of LV inflow by transmitral VTI and mitral annulus dimension is highly error-prone

*AR* aortic regurgitation, *CW* continuous-wave, *EROA* effective regurgitant orifice area, *2D-PISA* *method* two-dimensional proximal isovelocity surface area method, *LV* left ventricular, *LVOT* left ventricular outflow tract, *MR* mitral regurgitation, *PW* pulsed wave, *RVOT* right ventricular outflow tract, *VTI* velocity time integral

The echocardiographic parameters of the integrative approach are influenced by the individual hemodynamic conditions, by anatomical specifics, and by methodological factors. Therefore, errors and misjudgments are possible. In addition, the existence of multiple true jets impedes the grading of AR and MR severity. However, in many cases, the finding of ‘multiple jets’ is the result of the echocardiographic cut-plane displaying segments of an elliptic, crescent-shaped, or non-circular geometric regurgitant orifice area (GROA). This is commonly seen in secondary MR, but also in AR, especially in patients with a bicuspid AV. In the presence of multiple jets, biplane assessment of the vena contracta (VC) can be used. However, there are no established cut-offs for this biplane assessment of combined MR and AR.

Severe discordances between echocardiography and cardiac magnetic resonance imaging (MRI) for grading MR severity were recently found [9, 10, 41]. In addition, the debatable data were reported in trials of interventional MR therapy for LVSV_tot_ and regurgitant volume through the MV (RegVol_MR_), documenting low flow conditions which are not compatible with live conditions [42–44], reveal the weakness of the echocardiographic integrative approach, if plausibility of hemodynamics is not considered. Therefore, a severe underestimation of LV end-diastolic volume (LVEDV) and overestimation of RegVol_MR_ can be assumed in these trials [45–48]. Similar errors in grading AR and MR severity are probable in routine settings—especially because qualitative or semi-quantitative grading of AR and MR severity by jet area and the two-dimensional proximal isovelocity surface area (2D-PISA) method are still commonly used [49].

In this paper, we discuss the echocardiographic integrative approach in order to find evidence to support a quantitative approach for grading AR and MR severity [18, 50, 51]. There is still skepticism as to whether cardiac volumes can be accurately determined by echocardiography because several studies have reported differing cardiac volumes when measured by echocardiography or cardiac MRI [52, 53]. This is surprising because methodological studies using phantoms have shown comparable volumes between the two methods [54]. Contour delineation of the inner edge due to blurring underestimates volumes in the range of 5–10%, even in phantoms [52]. The pronounced differences in LV volume determination when using different methods are therefore incomprehensive, inconclusive, and contradictory [55, 56]. Plausible explanations for lower cardiac volumes by native 2D echocardiography in comparison with 2D contrast and native 3D echocardiography as well as with cardiac MRI in the clinical setting [55, 57, 58] are methodological errors due to foreshortening or differing contour delineations of the endocardium and limitations due to spatial resolution. In contrast to these previous studies, recent communications and trials using modern ultrasound technologies showed that comprehensive echocardiography can provide reliable and verifiable cardiac volume measurements by planimetry/volumetry as well as by Doppler echocardiography to correctly characterize cardiac hemodynamics [18, 59–62]. In conclusion, using a definitive quantitative approach to grade the severity of valvular regurgitation includes the acceptance of the requirements to properly and plausibly determine LV volumes by echocardiography. Based on this assumption normal values, cut-off values of LVEDV, LV end-systolic volume (LVESV), regurgitant volumes and regurgitant fraction (RF) are provided in recommendations and guidelines for the echocardiographic assessment of valvular regurgitation [3, 7, 8, 11, 17, 20].

Concerning the quantitative assessment in isolated valvular AR or MR, similar cut-offs for regurgitant volume (≥ 60 mL) as well as RF (≥ 50%) have been defined for severe regurgitations.

**Fig. 1:**
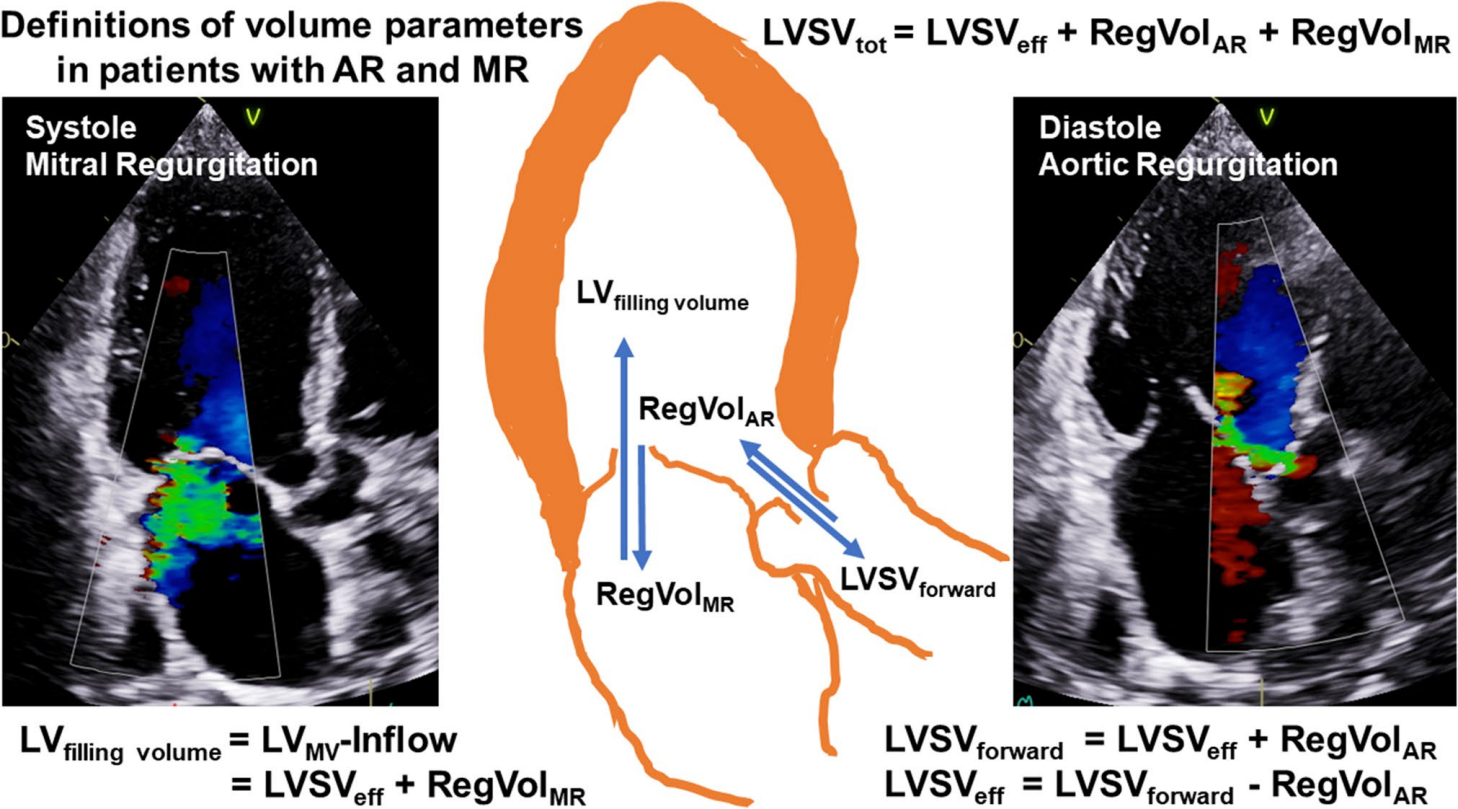
Definition of LV filling volume (LV_filling volume_), LV forward stroke volume (LVSV_forward_), total and effective LV stroke volume (LVSV_tot_, LVSV_eff_). *RegVol*_*MR*_ regurgitant volume through the mitral valve, *RegVol*_*AR*_ regurgitant volume through the aortic valve

**Fig. 2:**
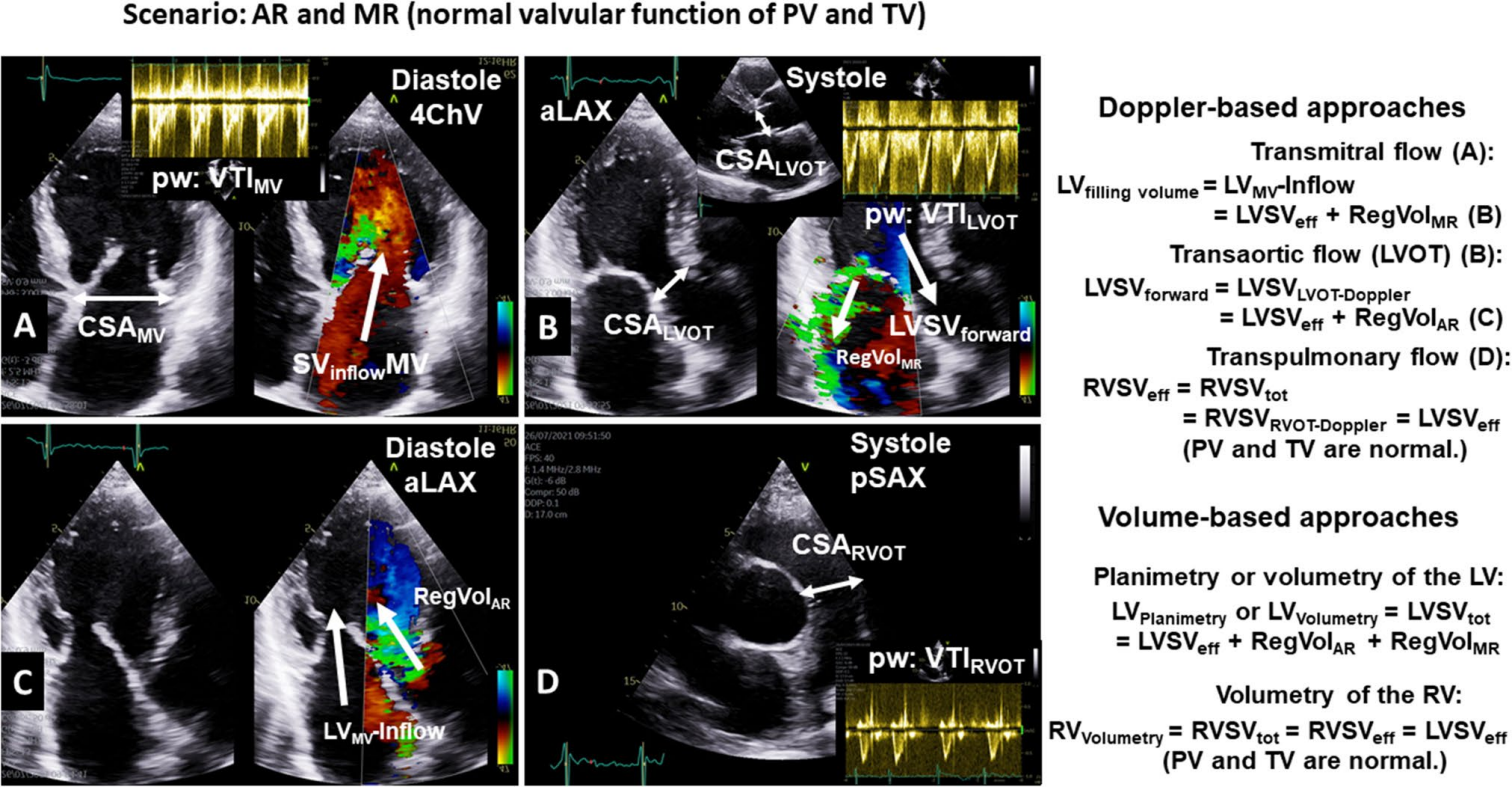
Definition of volumes determined by Doppler echocardiography and planimetry or volumetry by 2D echocardiography in patients with combined AR and MR and normal pulmonary and tricuspid valve. *AR* aortic regurgitation, *CSA* cross-sectional area, *LVOT* left ventricular outflow tract, *MR* mitral regurgitation, *MV* mitral valve, *RVOT* right ventricular outflow tract, *PW* pulsed-wave Doppler, *VTI* velocity time integral, *aLAX* apical long-axis view, *pSAX* parasternal short-axis view, *4ChV* 4-chamber view

Considering the methodological limitations of Doppler echocardiography and low-flow conditions in heart failure patients with secondary MR, a rigid cut-off of 60 mL for severe regurgitation might prove impractical in individual patients—especially when dealing with interdependent valve lesions [3, 7, 8, 11, 17, 20]. Therefore, a cut-off value of ≥ 45 mL for severe secondary MR has been proposed in low-flow conditions [20]. Consequently, when assessing the hemodynamic impact and relevance of combined AR and MR in a setting where each valve lesion seems only moderate, quantitative echocardiographic assessment should focus on estimating the total as well as each individual RF. Understanding the definition of the respective LV volumes—particularly LV filling volume, LVSV_tot_ and LVSV_eff_—and their echocardiographic assessment is a prerequisite for reliably assessing hemodynamics in patients with combined AR and MR (see Fig. 1). Thus, the RegVol_AR_ and RegVol_MR_ can be estimated using different approaches (see Fig. 2).

### Doppler- and volumetry-based approaches to assess the volumes of the left and right ventricle

In general, cardiac volumes—filling volumes, stroke volumes and regurgitant volumes—can be determined by different echocardiographic methods. Both Doppler techniques and volumetric measurements have methodological limitations. For example, when using pulsed-wave (PW) Doppler, it is essential to align the cursor with the position of the sample volume; when using planimetry or volumetry, labeling the mitral annulus and making a delineation between compacted and non-compacted myocardium make an accurate assessment challenging.

1. Usually, 2D planimetry or 3D volumetry of the left ventricle enables the measurement of LVSV_tot_. LVSV_tot_ is determined using either the biplane Simpson’s method of discs, triplane planimetry, or 3D volumetry by subtracting LVESV from LVEDV.
2. Only 3D volumetry enables the measurement of total RV stroke volume (RVSV_tot_). However, RV trabecularization and the complete acquisition of the anterior RV wall within the 3D dataset are methodologically challenging. Thus, in the presence of normal pulmonary and tricuspid valves without regurgitation, LVSV_eff_ can be determined using 3D RV volumetry. Under these circumstances, effective RV stroke volume (RVSV_eff_) corresponds to RVSV_tot_.
3. The mathematical basis of cardiac flow quantification by Doppler echocardiography in the absence of stenoses and regurgitation at all cardiac valves as well as shunts is that RVSV_eff_ is equal to LVSV_eff_, labeled as the ratio between pulmonary and systemic flow (*Q*_p_/*Q*_s_).
4. LVSV_eff_ represents the LV stroke volume, which is responsible for the arterial blood flow within the circulation. In the absence of AR, LVSV_eff_ corresponds to LVSV_forward_. If AR is present, LVSV_eff_ corresponds to LVSV_forward_ − RegVol_AR_. In addition, LVSV_eff_ in patients with AR and MR can be calculated using the equation LVSV_eff_ = LVSV_tot_ − RegVol_AR_ − RegVol_MR_.
5. LVSV_forward_ is given by the equation LVSV_forward_ = CSA_LVOT_ × VTI_LVOT_. The cross-sectional area (CSA) of the LVOT (CSA_LVOT_) is calculated by CSA_LVOT_ = π × (*D*_LVOT_/2)^2^, where D_LVOT_ corresponds to the diameter of the LVOT, which is usually determined in the parasternal long-axis view due to the best possible spatial resolution. VTI_LVOT_ is the velocity time integral (VTI) determined at the level of the LVOT using PW Doppler.
6. By analogy, RVSV_eff_ corresponds to forward RV stroke volume (RVSV_forward_) if no pulmonary regurgitation is present. In addition, in the absence of tricuspid and/or pulmonary regurgitation, RVSV_eff_ represents total RV stroke volume (RVSV_tot_). However, data to confirm the feasibility and reliability of assessing right-sided RVSV_eff_ in the setting of combined AR and MR are still lacking.
7. RVSV_forward_ is given by the equation RVSV_forward_ = CSA_RVOT_ × VTI_RVOT._ CSA_RVOT_ is calculated by CSA_RVOT_ = *π* × (*D*_RVOT_/2)^2^, where *D*_RVOT_ is the diameter of the RV outflow tract (RVOT) determined in the parasternal or subcostal short-axis view. VTI_RVOT_ is determined at the level of the RVOT using PW Doppler. Due to the tapered shape of the RVOT, it can be helpful to measure RVSV_eff_ at the level of the circular CSA of the pulmonary valve or the proximal pulmonary trunk in combination with the actual positions of the PW sample volumes, respectively. However, data to confirm the feasibility and reliability of assessing right-sided RVSV_eff_ in combined AR and MR are still lacking.
8. The transmitral LV volume inflow (LV_MV_-Inflow) describes the LV filling volume and can be estimated by the equation LV_MV_-Inflow = CSA_MV_ × VTI_MV_ or = CSA_MA_ × VTI_MA_. The cross-sectional area (CSA) of the MV orifice (CSA_MV_) or of the MA is calculated by CSA_MA_ = *π* × (*D*_MV_/2)^2^ or by CSA_MA_ = *π* × (*D*_MA_/2)^2^, where *D*_MV_ and *D*_MA_ correspond to the diameters of the MV orifice and MA, which are usually determined in the apical 4-chamber view. However, due to the non-circular shape of the MV orifice area and the mitral anulus, the calculation of LV_MV_-Inflow using Doppler echocardiography is highly error prone. Planimetry of the respective MV levels using 3D echocardiography may compensate this anatomical challenge. In either approach, the PW Doppler sample volume must be carefully positioned at the corresponding level vor proper calculation of CSA_MV_ and CSA_MV_, respectively. In theory, the sum of LV_MV_-Inflow and RegVol_AR_ corresponds to LVSV_tot_. In addition, LV_MV_-Inflow corresponds to the sum of LVSV_eff_ and RegVol_MR_.
9. RegVol_AR_ can be determined using the 2D-PISA method which is rarely feasible. However, this approach is suitable only if the proximal convergence area is perpendicular to the alignment of the cursor and in the center of the jet formation. Alternatively, RegVol_AR_ can be calculated by subtracting RVSV_eff_ from LVSV_forward_, which seems to be more practical than an assessment of RegVol_AR_ by 2D-PISA.
10. RegVol_MR_ can be determined using the 2D-PISA method. However, 2D-PISA in MR has known limitations [8, 18]. Among other reasons, eccentric jet formations in primary and secondary MR as well as small left ventricles cause significant errors due of RegVol_MR_. Alternatively, RegVol_MR_ can be calculated by subtracting LVSV_tot_ from LVSV_forward_ in patients with AR and MR. However, this approach is methodologically challenging due to potential underestimation of LVSV_tot_ by 2D planimetry or 3D volumetry.
11. Both RegVol_MR_ and RegVol_AR_ do not contribute to the effective circulation or LVSV_eff_. Since the regurgitations appear sequentially in the cardiac cycle, i.e., RegVol_MR_ during systole and RegVol_AR_ during diastole, the addition of the regurgitant volumes RegVol_AR_ and RegVol_MR_ would yield the overall or total regurgitant volume (RegVol_tot_) in patients with combined AR and MR.
12. In conclusion, total RF (RF_tot_) can be calculated using the equation (RF_tot_) = (RegVol_AR_ + RegVol_MR_)/LVSV_tot_. Although theoretically meaningful, there are many limitations owing to the measurements used, the circumstances of the measurements, and the interdependence of these lesions in an individual patient. Finally, the pathophysiological and clinical impact of RF_tot_ ≥ 50% due to combined moderate AR and MR may not be equivalent to RF ≥ 50% of one single severe lesion.
13. The assessment of LVSV_forward_ and RVSV_forward_ offers yet another intriguing plausibility check to estimate individual RegVol_AR_ and RegVol_MR_, since it examines the problem of combined AR and MR using principles similar to those applied for assessing intracardiac shunting based on the ratio of RVSV_eff_/LVSV_eff_ (known as *Q*_p_/*Q*_s_ ratio). Hemodynamic relevance of combined AR and MR can be assumed when the RVSV_eff_/LVSV_eff_ ratio is ≤ 0.74, equaling RF at the AV (RF_AR_) ≥ 35% when RVSV_eff_ = LVSV_eff_ = LVSV_forward_ − RegVol_AR_.
14. The assessment of volume parameters is extremely difficult in patients with atrial fibrillation due to beat-to-beat variations. Therefore, averaging of multiple measurements is recommended.

### Potential improvements to characterize AR and MR severity in multiple VHD by echocardiography

Both AR and MR tend to increase RV and PA pressures. Both lesions increase LV size, which in turn increases functional MR but almost never AR. Increased LVEF is recognized as a suitable due to AR contributes to LA and mitral annulus dilatation and thus to functional MR. LV dilatation caused by severe AR [7, 8, 20] must be critically verified in the setting of combined moderate AR and MR. An increased LV preload owing to the additional MR causes more severe and possibly earlier LV dilatation than LV dilatation caused by AR alone [37]. However, in the context of secondary MR, LV dilatation may also be caused by entirely other diseases like dilated or ischemic cardiomyopathy or myocarditis. In these scenarios, AR may only be a bystander. One possible way to discriminate between these two pathophysiological entities is to examine the ratio of leaflet area to annulus area, which is higher in patients with both significant AR and MR [37, 38]. Since both AR and MR increase LV preload and AR increases LVEDP while decreasing LV compliance, exercise echocardiography might be useful in assessing combined AR and MR. While also exposing typical symptoms, exercise testing can induce an increase in PA pressure with values ≥ 60 mmHg indicating significant hemodynamic relevance in combined AR and MR [63].

**Fig. 3:**
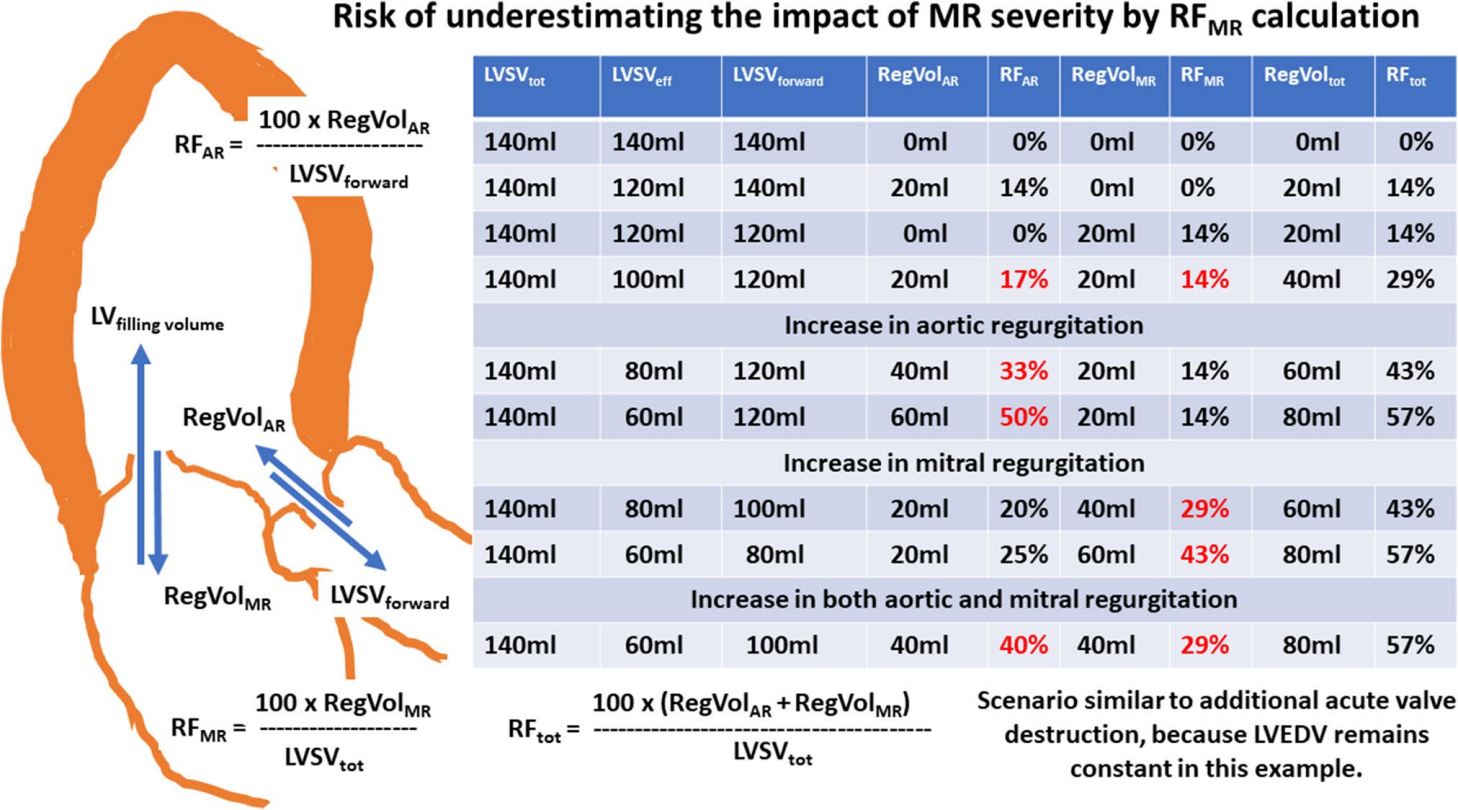
Juxtaposition of total and effective LV stroke volume (LVSV_tot_, LVSV_eff_), LV forward stroke volume (LVSV_forward_), regurgitant volume through the aortic, mitral valve and both valves (RegVol_AR_, RegVol_MR_, RegVol_tot_) as well as regurgitant fraction at the aortic and mitral valve (RF_AR_, RF_MR_) and total regurgitant fraction of both defects (RF_tot_). The numerical example represents acute alterations as seen in valvular destruction due to endocarditis without chronic compensation mechanisms. The impact of MR severity by conventional assessment is underestimated in comparison to AR severity (see red numbers). *AR* aortic regurgitation, *MR* mitral regurgitation

**Fig. 4:**
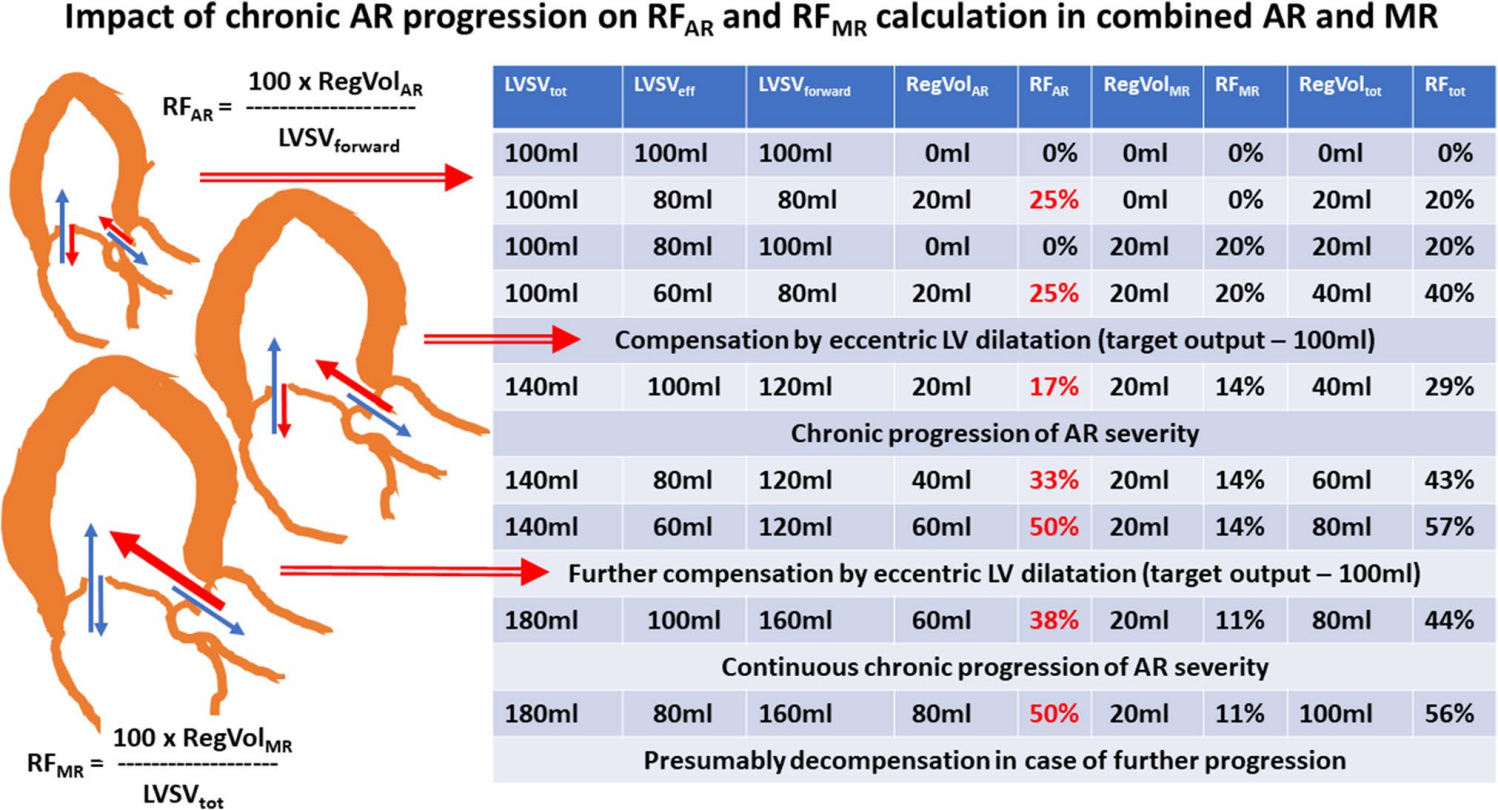
Juxtaposition of LVSV_tot_, LVSV_eff_, LVSV_forward_, RegVol_AR_, RegVol_MR_, RegVol_tot_, RF_AR_, RF_MR_ and RF_tot_. with progression of chronic AR (see red numbers) in the presence of MR. Abbreviations: see Fig. 3

**Fig. 5:**
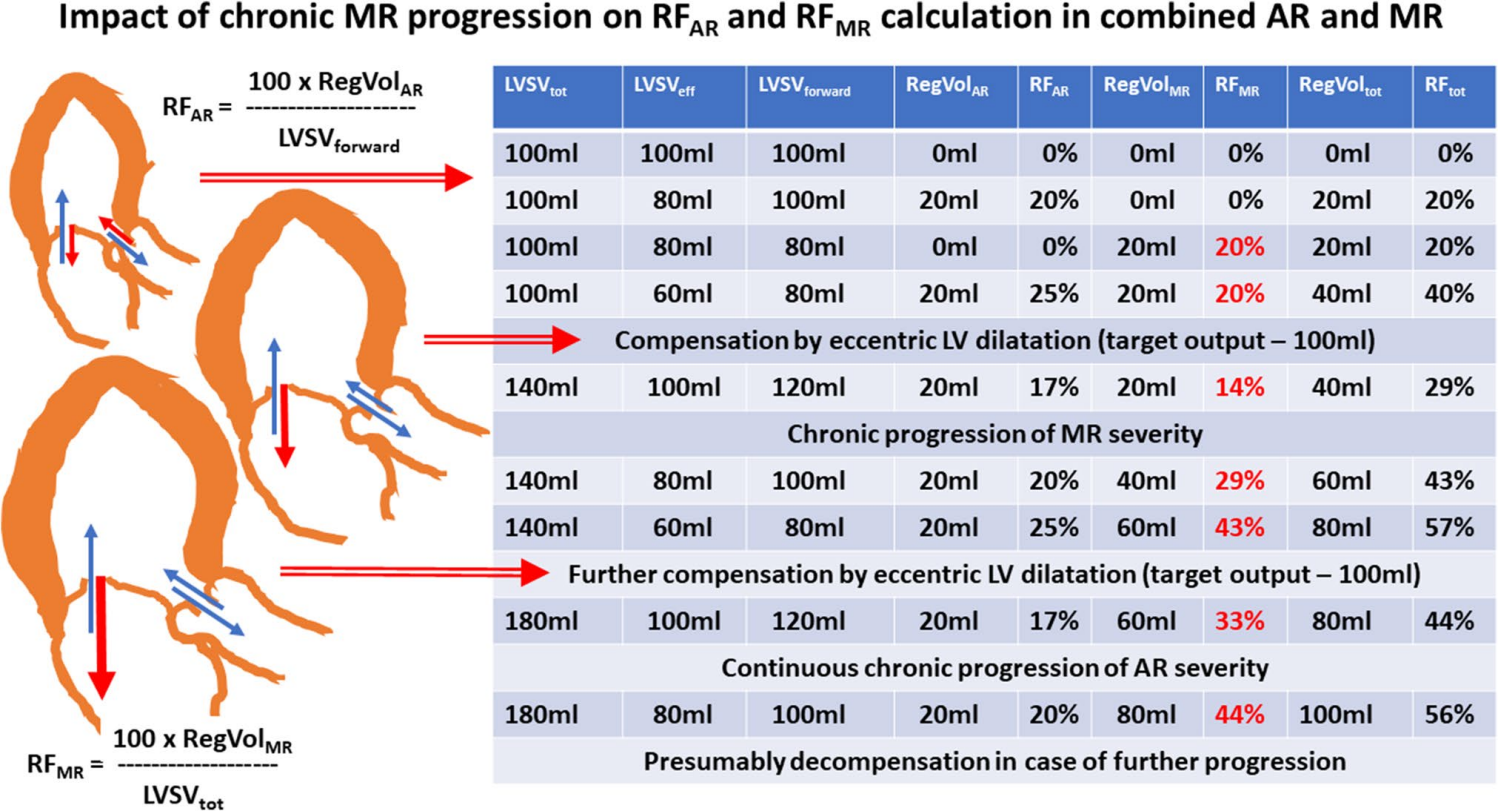
Juxtaposition of LVSV_tot_, LVSV_eff_, LVSV_forward_, RegVol_AR_, RegVol_MR_, RegVol_tot_, RF_AR_, RF_MR_ and RF_tot_. with progression of chronic MR (see red numbers) in the presence of AR. Abbreviations: see Fig. 3

**Fig. 6:**
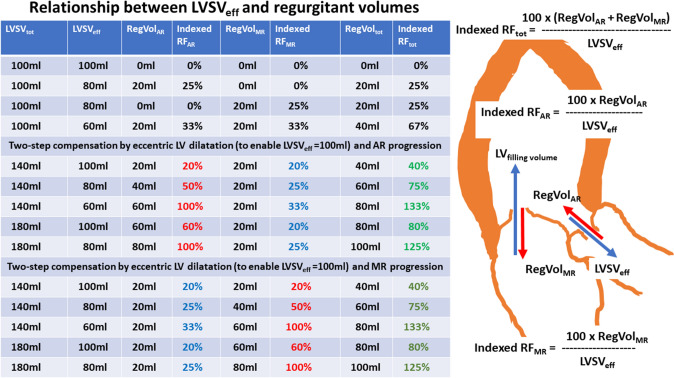
Relationship between LVSV_eff_ and RegVol_AR_, RegVol_MR_, and RegVol_tot_ as well as the corresponding indexed RF_AR_, indexed RF_MR_, and indexed RF_tot._ Juxtaposition of these values with progression of chronic AR or MR. Abbreviations: see Fig. 3

A general problem when assessing regurgitant volumes and regurgitant fractions is the reference size of the single valvular regurgitation. Whereas the ratio of RegVol_AR_ to LVSV_forward_ determines RF_AR_, the ratio of RegVol_MR_ to LVSV_tot_ characterizes RF_MR_. Thus, RF_AR_ is comprehensibly analyzed with varying regurgitant volumes through the AV, because an increase in RegVol_AR_ will cause an increase in LVSV_forward_. In contrast, the relevance of RF_MR_ cannot be properly assessed by RegVol_MR_/LVSV_tot_, because RF_MR_ remains stable with an increasing amount of RegVol_AR_. Theoretically, despite a decrease in LVSV_eff_ due to an increase of RegVol_AR_ (assuming a constant LV size), RF_MR_ can be stable. An increase in RegVol_MR_ (assuming an increasing LV size) RF_MR_ can be stable, too (see Figs. 3, 4, 5). Consequently, the contribution of RegVol_MR_ and RF_MR_ to the individual hemodynamic scenario in combined MR and AR can be surprisingly misinterpreted. Therefore, the determination of RegVol_tot_ to characterize the impact of both MR and AR might be more meaningful. In addition, it might be expedient to relate the individual regurgitant volumes of both MR and AR to LVSV_eff_ for a better characterization of their individual impact on hemodynamics (see Fig. 6). However, the ranges of the values for indexed RF_AR_ and indexed RF_MR_ as well as indexed RF_tot_ differ to the conventional values of RF_AR_, RF_MR_ and RF_tot_. The indexed RF values do not differ with respect to comparable amounts of regurgitant volumes at the respective valves. In addition, use of these indexed RF values is not yet introduced and implemented in current recommendations.

### Therapeutic considerations in combined AR and MR

Echocardiographic assessment can be used to define the predominant lesion and underlying mechanism of the lesion as well as estimate the potential treatment implication for the predominant lesion. It puts the focus on anticipating whether or not the treatment of one singular lesion can improve the symptoms and/or the functional state of the other lesion.

In moderate and severe AR, at least moderate MR is present in 5–45% [6, 27, 30, 37]. In the study of Lim et al., 35% of patients undergoing isolated AV replacement due to severe AR had concomitant moderate functional MR [64]. In 88% of these patients, MR improved to mild functional MR associated with postoperative LV remodeling [64]. However, the current evidence is conflicting [65]. Another study showed lower survival rates for surgical AV replacement alone compared to the combined treatment of significant AR and at least moderate primary or secondary MR—especially when MV repair was feasible [27]. Furthermore, in patients with relevant combined AR, MR will most likely be secondary. However, primary MR is present in up to 5% of combined moderate or severe AR and MR, favoring concomitant surgical therapy [27]. Considering that significant MR has less or even little impact on AR severity [7, 8, 17, 20] simultaneous treatment of both AR and MR is understandable.

The current database of the Society of Thoracic Surgeons (STS) reveals an increasing number of concomitant aortic and mitral valve surgeries over the last years [66]. This may be due to advances in the surgical technique and growing experience in the perioperative setting. However, the morbidity and mortality of concomitant valvular surgery must still be taken into account [67, 68]. Thus, the decision to undergo surgical or interventional therapy remains challenging. The following factors should be considered:

1. Individual operative surgical risk for the patient: Simultaneous surgical treatment of AR plus MR significantly increases the surgical risk for the patient [69–72]. The surgical risk must be balanced against the outcome for the patient with isolated correction of single valvular regurgitation. This decision is critical in older patients who may not experience the complications caused by the second valvular dysfunction [71, 72].
2. Severity of regurgitation of the non-dominant valve lesion: Usually, regurgitation of a single valve is the leading cause of symptoms. Concomitant regurgitation of the non-dominant valve lesion is often of minor degree. This constellation may influence the decision whether a complete repair of both valves is preferable.
3. Possibility of mitral repair versus mitral replacement: In isolated degenerative MR, repair is associated with a lower mortality and better long-term outcome than MV replacement [73]. Thus, the current guidelines recommend MV repair where feasible [20]. Outcome data in recent registries show an increased mortality in patients with single AV or MV replacement compared to combined AV replacement and MV repair [68]. Although these results may be caused by confounders, repair remains the less invasive procedure and should therefore be given preference where feasible.
4. Potential of spontaneous improvement of MR: As outlined above, MR may improve significantly after AV replacement [64]. This potential therapeutic sequela should be considered in secondary MR and significant LV dilatation due to AR.
5. Suitability for transcatheter mitral edge-to-edge repair (TEER): Interventional MV therapy may be considered in inoperable patients with isolated primary and secondary MR [20]. The intervention can be performed with a low periprocedural risk [42–44]. A approach with primary AV replacement and secondary transcatheter intervention may be appropriate—especially in combined AR and MR patients with a high surgical risk and favorable MV anatomy. As transcatheter therapies for structural heart diseases continue to develop, combined AR and MR might be treated sequentially with a low interventional risk in future [74].

In summary, the decision how to treat combined AR and MR is complex. Thus, it should be made by a team including cardiac surgeons, interventional cardiologists, and cardiovascular imaging specialists.

### Practical guide for implementing the quantitative approach in combined AR and MR patients

Significant individual variations in the LV volume despite a good correlation have been described for 2D planimetry/3D volumetry and Doppler echocardiography in the literature [56]. However, recent studies using Doppler echocardiography show no differences in cardiac output in comparison to thermodilution [60]. In addition, using modern techniques—particularly real-time 3D echocardiography—no significant or only minor differences in cardiac volumes are described in comparison with MRI [75, 76]. Thus, implementing new echocardiographic technologies in routine settings will presumably enable quantitative cardiac volume assessment in future, provided that echocardiography is performed correctly.

1. The first step in using the quantitative approach in VHD patients is a self-testing of the measuring procedures in controls, where identical values of LVSV_eff_ = RVSV_eff_ = LVSV_tot_ = RVSV_tot_ = LVSV_forward_ = RVSV_forward_ must be determined. If verifiable values cannot be determined, the quantitative approach should not be used.
2. In general, methodological aspects to avoid volume underestimation by 2D planimetry or 3D volumetry and volume overestimation by Doppler echocardiography and 2D-PISA should be considered. Nevertheless, even experienced investigators can make mistakes which must be corrected by improved image documentation and consecutive plausibility checks.
3. The ‘red flags’ of Doppler echocardiography can be qualitatively used to estimate AR and MR severity. However, grading according to jet area is not one of the ‘red flags’ and is definitely not recommended.
4. RegVol_AR_ is best quantified by comparing LVSV_forward_ and RVSV_forward_ using the equation RegVol_AR_ = LVSV_forward_ − RVSV_forward_.
5. RegVol_MR_ is best quantified by comparing LVSV_tot_ and LVSV_forward_ using the equation RegVol_MR_ = LVSV_tot_ − LVSV_forward_.
6. The main goal of the quantitative approach, however, is to identify the dominant valve lesion.
7. If echocardiography does not provide for a plausible hemodynamic interpretation, MRI should additionally be considered.
8. In any case, the multimodal imaging approach supports correct decision making as regards therapy.

## Summary and conclusion

Interpreting the hemodynamic scenario in combined AR and MR is more challenging than in pathologies caused by single regurgitation. As a result, the assessment of combined AR or MR severity remains difficult. Grading of AR and MR severity by ‘eyeballing’ and/or by 2D-PISA is often used in clinical practice but is generally misleading due to inconsistent results. Due to the uncertainty of a reliable assessment using some compounds of the integrative approach, the focus should be on performing a quantitative analysis of the respective severity of each regurgitation and of the RegVol_tot_ and RF_tot_ in combined AR and MR. Verifiable documentation is a prerequisite for a quantitative assessment by echocardiography to ensure accurate and plausible measurements of cardiac volumes. The present proposal provides a detailed systematic approach to understand the underlying pathophysiology and address the diagnosis and severity evaluation in patients with combined AR and MR. Nevertheless, the diagnostic goal of detecting a severe impact of both lesions on hemodynamics—with its therapeutic implications—remains challenging, especially when each valve lesion seems only moderate. Thus, future studies are necessary to obtain more data on the interactions of combined AR and MR for adequate therapeutic decision making.

## Data Availability

Data sharing is not applicable to this article as no new data were created or analyzed in this expert proposal.

## Appendix: Conference discussion

*Prof.* *Dr.* *Andreas* *Hagendorff* *(Leipzig):* One major topic at the “Deutscher Echokardiographie Kongress 2022” in Leipzig was multiple VHD. The present expert proposal focuses on a specific combination—the simultaneous presence of AR and MR. To start off the conference discussion:

How often do you see the combined entity of AR and MR? How often is this scenario clinically relevant?

*Prof.* *Dr.* *Fabian* *Knebel* *(Berlin):* The coexistence of two valvular lesions in transthoracic echo is a frequent finding. However, in most cases, there is a dominant lesion. The coexistence of two equally relevant lesions is seen only in a minority of patients. My strategy with regard to imaging in these cases is to perform transthoracic echocardiography (TTE) (preferably without sedation to avoid influencing hemodynamics)—it is only after TTE and transesophageal echocardiography (TEE) that I can clearly quantify the severity of each lesion.

*Prof.* *Dr.* *Andreas* *Hagendorff* *(Leipzig):* Describe the most important scenario of acute combined AR and MR. In what situation does the occurrence of AR aggravate mild or moderate MR and vice versa?

*Elena* *Romero-Dorta* *(Berlin):* The presence of acute combined AR and MR can be expected—almost exclusively—in the setting of endocarditis, which induces valvular destruction. Acute valvular dysfunction is not accompanied by compensatory mechanisms such as LV remodeling. Therefore, it presents with acute relevant symptoms. Nevertheless, drastic worsening of concomitant chronic AR and MR is also likely when the compensation limits for regurgitant volumes of both valves are reached. Concomitant AR and MR primarily lead to LV volume overload. While a functionally intact MV prevents impairment of the low-pressure system in chronic AR, the coexistence of MR accelerates hemodynamic deterioration and worsens the prognosis of the patient by causing pulmonary hypertension, RV dilation and RV dysfunction, among others. With this in mind, it also makes sense to think from a pathophysiological point of view that occurrence of acute AR, for example due to aortic dissection, may be less tolerated in patients with concomitant chronic MR.

*Prof.* *Dr.* *Andreas* *Hagendorff* *(Leipzig):* Eccentric LV hypertrophy with LV dilatation is a compensatory mechanism in chronic AR and/or MR. Progression of which type of regurgitation—AR or MR—is more likely and more relevant in the clinical setting?

*Dr.* *Aydan* *Ewers* *(Bochum):* LV dilatation due to eccentric LV hypertrophy and/or other causes, for example cardiomyopathy, can primarily cause MA dilatation. The MA is formed—particularly in the posterolateral regions—by converging muscular fibers of the atrial and ventricular myocardium. Therefore, MA dilatation due to LV dilatation is more likely than aortic annulus dilatation. In contrast, AR progression due to diverging cusps is more likely with aortic root dilatation than with LV dilatation.

*Prof.* *Dr.* *Andreas* *Hagendorff* *(Leipzig):* Should we wait for symptoms to occur in patients with combined AR and MR? Or should we assess the hemodynamics for decision-making prior to symptoms occurring? How can we handle this problem?

*Dr.* *Roland* *Brandt* *(Bad* *Nauheim):* In general, compensatory mechanisms in VHD are very effective. Consequently, if symptoms become manifest, a late stage of the disease is likely. Therefore, in some cases acute cardiac decompensation—especially due to malignant arrhythmias—may be the first symptom in severe VHD. It is obvious that treatment should be initiated prior to these events. Monitoring of hemodynamics by echocardiography in VHD patients at an early stage might be one solution to this problem.

*Prof.* *Dr.* *Andreas* *Hagendorff* *(Leipzig):* What echocardiographic methods are predominantly used in the clinical setting? Is a qualitative estimation by ‘eyeballing’ jet areas or a semi-quantitative calculation based on the 2D-PISA method fair to our patients?

*Prof.* *Dr.* *Dariush* *Haghi* *(Ludwigshafen):* Qualitative ‘eyeballing’ of the jet area in patients with regurgitation is, of course, the most frequently used method in clinical routine, even if guidelines do not recommend using the jet area to estimate the severity of regurgitation. The often misleading and error-prone 2D-PISA method is the second most frequently used method. The classification of MR by echocardiography using the integrative approach and by quantitative MRI yields inconsistent results. It highlights the weakness of the echocardiographic integrative approach.

*Prof.* *Dr.* *Andreas* *Hagendorff* *(Leipzig):* Comment on the special limitations of the 2D-PISA method for the assessment of RegVol_AR_.

*Prof.* *Dr.* *Ertunc* *Altiok* *(Aachen):* The regurgitant orifice area in AR often has a non-circular shape and, in these cases, the 2D-PISA method should not be applied. Furthermore, measurements obtained with the 2D-PISA method may be inaccurate in patients with an obtuse flow convergence angle and/or wall-impinging flow convergence zone. Due to methodological limitations, the only AR entity for which 2D-PISA may be suitable is prolapse of the right coronary cusp using the parasternal approach.

*Prof.* *Dr.* *Andreas* *Hagendorff* *(Leipzig):* Do you think 3D echocardiography—and especially the visualization of the color-coded regurgitant orifice areas by color-coded 3D zoom datasets—would provide for a better assessment of AR and MR severity?

*Dr.* *Nicolas* *Merke* *(Berlin):* Modern 3D echocardiography with a sufficient temporal and spatial resolution is pivotal in the diagnostic work-up of the mechanism underlying AR and MR. 3D color datasets—especially 3D ZOOM—enable the analysis of the 3D regurgitant orifice area if the volume rate is sufficient and the settings avoid color pixel smoothing. Perhaps regurgitant flow analysis by 3D-PISA will be possible in the future. Using a reduced sector width with a small color box and multi-beat acquisition, the temporal resolution of 3D color signals can be improved. However, to my knowledge, generally accepted cut-off values for 3D regurgitant areas have not been published.

*Prof.* *Dr.* *Andreas* *Hagendorff* *(Leipzig):* Do you think the integrative approach for grading combined AR and MR severity is suitable for reaching a proper diagnosis? In your opinion, what are the main limitations of the integrative approach?

*Dr.* *Stephan* *Stöbe* *(Leipzig):* The integrative approach is a challenge even in patients with singular valvular regurgitation. The severity of regurgitation is often not accurately classified in comparison with a quantitative MRI assessment. Therefore, the integrative approach will presumably fail more often in combined AR and MR patients. The main limitations of the integrative approach are the still incomprehensible belief in the jet area philosophy as well as the wrong application and lack of methodological standardization of the 2D-PISA method.

*Prof.* *Dr.* *Andreas* *Hagendorff* *(Leipzig):* Will 3D echocardiography and artificial intelligence improve cardiac volume assessment by echocardiography?

*PD* *Dr.* *Christoph* *Sinning* *(Hamburg):* Using 3D echocardiography improves LV and RV volume measurements, enabling a better assessment of LV and RV remodeling in experienced laboratories. In addition, planimetry of the LVOT and RVOT area within 3D datasets often provides more reliable results than the area calculation using only one diameter. In conclusion, 3D volumes should generally be acquired in VHD patients to enable proper quantitative measurements by postprocessing.

*Prof.* *Dr.* *Andreas* *Hagendorff* *(Leipzig):* To focus on the quantitative approach, regurgitant volumes and regurgitant fractions are measured in patients with AR and MR using cardiac MRI. Why do we not do the same in echocardiography?

*PD* *Dr.* *Sebastian* *Ewen* *(Homburg/Saar):* The main problem is that there is still skepticism as to whether cardiac volumes can be determined accurately by echocardiography. However, there is clear evidence that echocardiography is able to generate comparable results to cardiac MRI using modern ultrasound technologies like real-time 3D echocardiography or contrast-enhanced echocardiography. In consideration of the limitations outlined in the manuscript and applied by the suggested systematic approach, echocardiography is able to adequately characterize AR and MR using the quantitative approach.

*Prof.* *Dr.* *Andreas* *Hagendorff* *(Leipzig):* There are differences between the calculation of RegVol_AR_ and RegVol_MR_. RegVol_AR_ is referred to as LVSV_forward_, RegVol_MR_ is referred to as LVSV_tot_. Is this potentially relevant for the individual patient?

*Dr.* *Stephan* *Stöbe* *(Leipzig):* The differences in the reference parameters to estimate the regurgitant volume in combined AR and MR patients can primarily result in underestimation of the degree of MR—especially if AR severity is underestimated by an only qualitative estimation. If RegVol_AR_ is relevant, LVSV_eff_ is significantly lower in combined AR and MR. Thus, the calculated RF_MR_ does not accurately reflect the hemodynamic situation. To avoid this mistake, we proposed the introduction of correspondingly indexed RF_AR_, indexed RF_MR_, and indexed RF_tot._

*Prof.* *Dr.* *Andreas* *Hagendorff* *(Leipzig):* RF_MR_ is reduced or underestimated despite an unchanged RegVol_MR_ with increasing LVEDV due to an increasing proportion of AR. Is there any way that echocardiography can improve our diagnostic approach?

*Dr.* *Tobias* *Ruf* *(Mainz)* The ‘red flags’ of Doppler echocardiography in our proposal is to differentiate between chronic LV dilatation due to chronic AR and LV dilatation in the presence of mild or moderate AR due to cardiomyopathy are very important. The targets to clarify the main cause of LV dilatation and to detect the dominant valvular lesion characterize the scenarios, in which mild or moderate MR becomes relevant.

*Prof.* *Dr.* *Andreas* *Hagendorff* *(Leipzig):* Is your decision to perform transcatheter MV therapy influenced by the presence AR and by AR severity?

*Dr.* *Tobias* *Ruf* *(Mainz)* Yes, due to the properties of fluids, the hemodynamic relevance of coexisting AR and MR is interconnected, which should be appreciated. In the setting of hemodynamical relevance, a general approach could be intervention of the AV, when a transcatheter approach is opted for, followed by re-assessment of the MV pathology in the later course. Of course, many exceptions could be discussed. For instance, when the AR is not dominant and the MR is based on primary, i.e., degenerative/structural origin, transcatheter therapy of first the MV could be a better option, again, when surgery is off the table. However, these examples foremost underline the need for the Heart Team to properly understand the patient and the underlying pathologies, ultimately making the best decisions possible.

*Prof.* *Dr.* *Andreas* *Hagendorff* *(Leipzig):* A short comment on the surgical treatment in combined AR and MR—do you always repair or replace both valves? In what scenarios is it necessary to repair only one valve?

*Dr.* *Jan* *Knierim* *(Berlin):* As mentioned previously, the surgical treatment of combined AR and MR is complex. The potential of reverse LV remodeling is a crucial point in decision making. A decrease in LV size may occur in AR patients after AV repair, especially if the duration of disease is short. Under these conditions, mild or moderate MR can disappear. In contrast, if myocardial function is impaired and the LV is severely dilated, treating both valves may be the better decision. However, LV dysfunction increases the surgical risk.

*Prof.* *Dr.* *Andreas* *Hagendorff* *(Leipzig):* To finalize our discussion—what is the most important message of the present proposal? If you were to make a recommendation, when would you send a patient for a comprehensive quantitative echocardiography?

*Dr.* *Andreas* *Helfen* *(Lünen):* The most important message of our proposal is that there is a need for accurate and standardized echocardiography to facilitate the difficult quantitative approach in combined AR and MR patients. Despite our practical guide the quantitative approach is not suitable for everyone. It needs plausibility controls by counter-checks and, if possible, a direct comparison with MRI to enable a better learning curve. It is time-consuming and challenging. It is not a tool for a rough orientation by echocardiography, but rather a tool for specialists in comprehensive echocardiography. This topic once again shows us the importance of teaching and training in echocardiography.
